# Mapping domains of early-life determinants of future multimorbidity across three UK longitudinal cohort studies.

**DOI:** 10.23889/ijpds.v9i5.2559

**Published:** 2024-09-18

**Authors:** Sebastian Stannard, Ann Berrington, Simon Fraser, Shantini Paranjothy, Rebecca Hoyle, Rhiannon Owen, Ashley Akbari, Mozhdeh Shiranirad, Roberta Chiovoloni, Nisreen Alwan

**Affiliations:** 1 School of Primary Care, Population Sciences and Medical Education, Faculty of Medicine, University of Southampton; 2 School of Economic, Social and Political Sciences, University of Southampton; 3 School of Medicine, Medical Sciences and Nutrition, University of Aberdeen; 4 School of Mathematical Sciences, University of Southampton; 5 Population Data Science, Swansea University Medical School, Faculty of Medicine, Health & Life Science, Swansea University; 6 University Hospital Southampton NHS Foundation Trust

## Objectives

Many studies use a reductionist approach to isolate the influence of one factor in childhood on multimorbidity rather than consider the combined effect of wider determinants. We aimed to explore how potential multiple early-life determinants of multimorbidity can be audited and characterised across three UK cohort studies.

## Approach

We used the 1958 National Child Development Study (NCDS), the 1970 British Cohort Study (BCS70), and the Aberdeen Children of the 1950s Study (ACONF) to identify and categorise early-life variables that fit into 12 previously conceptualised domains of early-life determinants of multimorbidity. A data audit manually assigned variables into the domain they best represented. Principal component analysis (PCA) reduced the dimensionality of the data and structured variables into subgroups. Important PCA components defined as the component that contributed the greatest proportion of the overall variance were identified.

## Results

Seven domains were characterised by 74 variables in ACONF, ten domains by 143 variables in the NCDS, and twelve domains by 289 variables in the BCS70. PCA analysis reduced the dimensionality of ACONF variables from 74 to 41, from 143 to 73 in the NCDS, and from 289 to 149 in the BCS70. Important PCA components included maternal fertility histories, long-term illnesses, educational ability, ethnicity, housing status and parental-child interactions.

## Conclusions and Implications

Conceptualising the risk of future multimorbidity as lifecourse domains composed of multiple factors can help challenge the existing understanding of disease aetiology and inform strategies for the prevention of multimorbidity.

